# Patients’ perception of own efforts versus clinically observed outcomes of non-surgical periodontal therapy in a Norwegian population: an observational study

**DOI:** 10.1186/s12903-015-0037-3

**Published:** 2015-05-17

**Authors:** Jon F Vatne, Per Gjermo, Leiv Sandvik, Hans R Preus

**Affiliations:** Department of Periodontology, Institute of Clinical Odontology, Faculty of Dentistry, University of Oslo, POB 1109 Blindern, 0317 Oslo, Norway

**Keywords:** Periodontitis, Compliance, Oral hygiene, Smoking, Clinical study

## Abstract

**Background:**

Most periodontal intervention studies have focused on biomedical qualities like change in pocket depth and clinical attachment levels. Very few studies have described patient response in terms of how patients' general lives are affected by disease, treatment, and communication with therapy providers. Thus the aim of the present study was to investigate patient response to systematic periodontal information, motivation and treatment strategy (primary aim) by comparing the patients’ perception of own efforts and results with those clinically registered in a trans-sectional, observational study (secondary aim).

**Methods:**

One year after treatment of 184 patients, 152 completed a questionnaire covering aspects of received oral health information and instruction, expectations, communication with the therapeutic team, behavioral change, self-perceived outcomes and satisfaction.

**Results:**

More than 90% of the patients were satisfied with the interaction with the specialist team. 98% were satisfied with the information and instruction they had been given. 84% said that the information had been necessary to make them change their behavior towards better oral hygiene. Pain and discomfort, as well as bleeding were reduced substantially from before to after treatment, and 28 patients reported to have stopped smoking. In all questions regarding well-being there were statistically significant changes towards positive impact following therapy.

**Conclusions:**

Periodontal treatment, including customized information and education on the etiology and pathogenesis, prevention and treatment as well as maintenance of periodontal diseases resulted in a high degree of short- and long term compliance, and very good patient centered outcomes, which again had a positive impact on the patients’ satisfaction. The patient centered outcomes correlated mostly with the compared clinical endpoints.

**Trial registration:**

ClinicalTrials.gov: NCT01318928.

## Background

Periodontal therapy has historically had a biomedical approach; with disease and health measured by clinical parameters like bleeding on probing (BOP), pocket depth (PD) and clinical attachment level (CAL) [1]. However, while disease belongs to the realm of biology, health also belongs to those of sociology and psychology, encompassing perceptions, feelings, behavior and ultimately quality of life [2]. Therefore, the last decades have procured an alternate breed of studies investigating the impact of disease and therapy on the patient’s quality of life by a bio-psychosocial approach to health in general [3], and to periodontal health in particular [4]. This research describes how patients’ general lives are affected by disease, treatment and communication with therapy providers. Wilson [5] emphasized that informing patients adequately causes increased compliance and reduced anxiety towards prospective therapy, emphasizing the need to assess these patient-related factors [5-11]. Hujoel [12] stated that periodontal research for decades have used surrogate endpoints for measurement of therapy. True endpoints should be tangible to the patient, evaluating how he feels and functions, including subjective quality-of-life measurements, patient satisfaction or self-reported symptoms as bleeding or pain from gums. Consequently, studies have shown that periodontal status is significantly associated with patient well-being [10,13].

The primary aim of the present study was therefore to evaluate patient response to a systematic periodontal treatment strategy by comparing the patients’ perception of their own efforts and results, with those clinically observed (secondary aim).

## Methods

**The study population** was recruited among referrals to a periodontal specialist clinic [14]. At the first visit all patients were presented with standardized written information on periodontal disease, its prevention and treatment and how behavior like oral hygiene and smoking affects its prevalence, development and recurrence. After a pre-treatment hygiene phase of approximately 3 months they were summoned for inclusion or exclusion for the study.

### Inclusion criteria

Age 35–75 years; ≥ 5 sites with a pocket depth ≥ 5 mm remaining after 3 months hygiene phase; no prior systematic periodontal treatment; no systemic diseases or medication known to be associated with periodontitis. Since metronidazole was to be included in the intervention of 2 of the study groups, the exclusion criteria also involved known allergies to, or adverse effects from this drug. Treatment commenced on 184 patients in March 2008, and 1-year follow-up completed in September 2010.

Upon inclusion, the patients were asked to sign an informed consent form, including verbal and written information that they would be subject to questionnaires, assisted as well as non-assisted, during the follow-up period of 5 years. At this point, oral hygiene was reinforced as needed, and the patients randomly allocated into 4 intervention groups using a random allocation table [15]. A codebook manager (PG) kept all patient and allocation data throughout the study - thereby securing blinding of the clinical research staff [14]. The four treatment strategies were [14]: Full Mouth Disinfection (FDIS) + Metronidazole (MET) (Group 1); FDIS + placebo (Group 2); Traditional, scaling and root planing (SRP) + MET (Group 3); and SRP + placebo (Group 4). Groups 1 & 2 received full mouth scaling and root planing (SRP) completed within a single workday (FDIS) using two sessions of 65 minutes each, two hours apart. In Groups 3 & 4 the SRP was completed using two 65-minute sessions, 21 days apart. Subsequent to all mechanical treatment sessions, in all groups, the patients rinsed for one minute with 10 ml 0.2% Chlorhexidine (CHX) (GlaxoSmithKline, Brentford, UK), and following mechanical instrumentation all sulci and pockets were filled with CHX gel (GlaxoSmithKline, Brentford, UK). In addition, patients in Groups 1 and 3 received MET (Sanofi-Aventis, Lysaker, Norway), 400 mg x 3 for ten days, starting the day before the two mechanical treatment sessions in Group 1 and the day before the second SRP session (day 20) in Group 3. Patients in Groups 2 and 4 received pharmacy-packed placebo tablets according to the same scheme as for Groups 1 & 3, respectively.

The study was designed as a randomized, double blind, clinical intervention trial comparing four intervention strategies, and was approved by the Regional Committee for Medical Research Ethics, South East Norway (2006/2012/245/REK). Clinical trial registration is http://www.clinicaltrials.gov – NCT01318928.

A locally adapted version of the motivational interviewing technique (MI) [16] was used throughout the study. In the interview, weight was put on both written and verbal information on periodontal disease before starting the instruction sequence. The interview always started with the question; “Do you know what periodontal disease is?” followed by a personally adapted explanation to each patient. Information on oral hygiene was designed with the aim that patients should never feel bad towards their own pre-study hygiene efforts. The word *hygiene* was deliberately omitted, and the patients were all told that the disease had changed their gingival anatomy to such an extent that their previous technique had become insufficient. Oral hygiene measures were shown in the mouth, first without and then with a mirror, emphasizing the ***feel*** of correct brushing in addition to visualization. During the hygiene phase, the patients were explicitly told that they would *not* receive treatment until they were able to clean their teeth to satisfaction.

### Informational strategy

A particular stringent way of communicating motivation placed a demand on the patient to do what he/she was being told. The education of the patient in all aspects of his/her disease; its etiology, pathogenesis, prevention, treatment, expected results and maintenance was elaborate and comprehensive – were given both verbally and in writing. The patient had to understand his/her condition, and all aspects around it, to accept the treatment and act according to instructions from the treatment team.

**Hypersensitivity** Patients reporting hypersensitivity at screening were treated in-clinic with fluoride varnish, given fluoride tablets and desensitizing dentifrice for home care. They were informed that, following treatment, it was customary to feel hypersensitivity for a period. This would be treated continuously, and especially at the 3 month post treatment follow-up, since the actual mechanical treatment (SRP/FDIS) is the activity that empirically is known to generate the most hypersensitivity.

**Pain and discomfort**, other than hypersensitivity, were addressed by thorough examination and diagnosis. Clinical procedures, other than periodontal, were performed by their referring dentist, specialists in endodontics or oral surgery prior to periodontal treatment (i.e. prior to baseline). Any complaint concerning pain or discomfort during or after the treatment phase was otherwise diagnosed and treated continuously.

**Smoking information** was provided as oral - and written information on how smoking is reported to affect periodontitis. The information was designed not to be condescending; containing no order, advice or suggestion to quit, and was accompanied by an offer of two weeks use of nicotine patches, free of charge. The main smoking information was given prior to baseline, followed up at treatment sessions as well as at the 3 and 12 months follow-ups. This information is compulsory in Norway in all periodontal treatment or - follow-up sessions - if refund from The Norwegian Health Economics Administration (HELFO) is required.

At screening, baseline, and 12 months post-therapy, PD and CAL was recorded in mm, and plaque and BOP as yes/no, in four sites of all teeth present. Smoking habit, medication and general health was revisited.

One year after active treatment, an informational letter and a questionnaire were mailed to the 180 patients, inviting them to participate – anonymity guaranteed - in this “patient satisfaction”- study. Six weeks later, a reminder was mailed to all 180 patients together with a copy of the questionnaire. Since the study focused on patient outcomes of periodontal treatment, a specific questionnaire was designed, also including relevant questions from the Oral Health Impact Profile (OHIP) [10,17-19]. Since it has been shown that compliance is the result of good communication and trust between the patient and the care provider [5-13], the first 11 questions were constructed to explore these issues. The next 11 questions explored pain and discomfort before and after treatment as well as the care and treatment that had been provided to that effect. Six questions explored the effect the treatment had had on the patients’ daily life, well-being, appearance, ability to chew, personal mood, social life and ultimately general feeling of health. Most questions had graded variables, from very bad to very good, whereas others were graded yes/no.

The study conforms to the Strobe guidelines for human observational studies, and underlying research material can be accessed at the project main website http://www.odont.uio.no/english/research/projects/periodontal-diseases/

### Statistics

Data were analyzed using the SPSS software, version 20. The study included 184 participants; 92 males and 92 females, who had been randomly allocated to 4 intervention groups [14]. Four patients left the study during the active treatment phase; one died; two were diagnosed with cancer, and one with diabetes mellitus, leaving 180 patients still in trial, and receiving the questionnaire, at the 12-month follow-up. For each question the frequency/percentages of scores were calculated totally as well as group-wise. Pearson’s Chi-squared test was applied to investigate significance of difference between scores as well as age or gender influence on behavior change and subjective symptom scores; student’s *t*-test for pairwise and independent samples; McNemar test to compare subjective symptom-scores before and after treatment (bleeding/pain, oral well-being and patient satisfaction). When analyzing change in the “patient satisfaction” variables from before to after treatment, a two-sided Wilcoxon signed-rank test was used. Significance level < 5% for all tests.

## Results

Totally 152 (84.4%) participants responded to the questionnaire; 61 (40%) said they knew and 79 (52%) said they did not know about their periodontal disease at the time of referral (12 (8%) non-responders).

### Satisfaction

Close to 99% (150) of the respondents were satisfied or very satisfied with the information and instruction they had received, and reported that the information had been essential to understand the planned treatment and maintenance (2 (1%) non-responders). There was no significant difference between genders or age groups.

More than 99% (151) were satisfied or very satisfied with the communication and interaction with the specialist team. One answered “not satisfied at all” (the opposite end of the scale of answers). Eighty-four per cent answered that the information they received had been necessary to make them change their “tooth-cleaning behavior”. The most commonly claimed behavior change was more efficient brushing (127 = 84%) together with more frequent interdental cleaning (131 = 86%), particularly the use of interdental brushes. One-hundred-and-one (66%) answered that they attended their dentists more often for maintenance. The clinically observed average percentage of sites with plaque in the *total* study population (184) changed from 61.9% at screening to 13.5% at inclusion following the described oral hygiene regime, and dropped even more throughout the first year post-treatment (Table 1).

**Table 1:** **Per cent surfaces with plaque (sd) in males and females compared to claimed improvement**

**Gender#**	**screening**	**Baseline (BL)**	**1 year**	p*(BL–1 year)	**Claimed improvement**		
						**Yes**	**No**
					**N**	**n (%)**	**n (%)**
**Males (n = 92)**	61.32 (10.71)	13.92 (14.17)	9.11 (14.37)	0.027	73	68 (93)	5 (7)
**Females (n = 92)**	60.64 (10.82)	11.29 (13.45)	5.68 (7.35)	< 0.001	77	57 (74)	20 (26)
p**	0.676	0.208	0.048		0.002***		

*Paired sample *t*-test.

**Independent samples *t*-test.

***Chi Square test (2 nonresponders).

# = total responders to the questionnaires from each gender.

Screening: Before hygiene phase.

Baseline: After hygiene phase, but prior to therapy.

1 year: 12 months after therapy.

PD and CAL (Table 2) as well as BOP (Table 3) were significantly changed for the better in all treatment groups, with no significant differences between groups or gender [14].

**Table 2:** **Clinical parameters; Pocket depth (PD), Clinical attachment level (CAL) and % patients with pockets (PD) > 5 mm before (baseline) and 12 months after treatment** [14]

**Parameter**	**Baseline**	**12 months after treatment**
Pocket depth in mm (sd)	3.09 (0.58)	2.24 (0.19)
Clinical attachment level mm (sd)	1.77 (0.99)	1.21 (0.78)
% patients with one or more sites withPD ≥ 5 mm	100	54

All significant (p < 0.05) changes from baseline to 12 months post therapy.

N = 184 at baseline, N’ = 176 at 12 months.

**Table 3:** **No of persons (%) with no BOP at baseline and 12 months after therapy according to gender**

**Gender**	**n**	**Screening**	**Baseline (%)**	**12 months (%)**
Male	92	0 (0%)	15 (16.3)	43* (48.8)
Female	92	0 (0%)	24 (26.1)	45* (51.1)

*n = 88 at 12 months (4 men and 4 women excluded).

Chi square baseline: p = 0.0957, at 12 m: p = 0.7655.

Chi square males: p = 0.00004, females: p = 0.0021.

### Patient centered outcomes

Prior to treatment, frequent or occasional gingival pain and hypersensitivity over time was reported by 123 (80%) responders. Following treatment, 143 (94%) experienced no - or negligible pain/discomfort; 7 (5%) reported more pain and discomfort than before treatment. (2 (1%) non-responders). This was mainly due to hypersensitivity, and more uncommonly excessive use/size of interdental brushes. Satisfaction with treatment results were reported by 144 (95%), and 146 (96%) said that they would like to be treated the same way again if necessary.

Prior to treatment, frequent or occasional bleeding from gums, was reported by 128 (84%) whereas 21 (14%) reported no such experience (3 (2%) non-responders). Following treatment, 12 (8%), 60 (40%) and 75 (49%) respectively, reported no difference; less and no more to experience bleeding. Of those that had reported frequent bleeding before treatment, 21 (52%) experienced less - and 18 (45%) experienced no bleeding after treatment whereas 1 (3%) reported no change. For comparison, positive BOP registration (at least 1 site with bleeding) for all at screening and 12 months following treatment went from 100% to 51% (Table 3).

### Differences between groups

No significant differences were found among intervention groups, age or gender, except that 68 (93%) of the males claimed subjective behavior change as they reported to brush more thoroughly after treatment. The same claim was done by 57 (74%) of the women (p < 0.05) (Table 1). The actual clinical registrations showed that although there was no gender-specific difference in percent sites with plaque at screening (before the hygiene phase), there was a great reduction in this parameter at the 12-month post-treatment control, with women scoring significantly better than men in all respects and periods (Table 1). There was no gender-difference regarding BOP at any time (Table 3).

### Smoking

Twenty-eight (18.4%) responders claimed to have quit smoking one year after treatment. For comparison; at baseline 92 patients were registered as current smokers, and on direct question from HRP at the one year clinical examination, 10 subjects claimed to have stopped smoking during this period. Seven patients had accepted the offer of free nicotine patches.

### Effect on “patient satisfaction” by treatment

In all questions regarding well-being (showing teeth when smiling, chewing ability, mood, social life and general health) there were significant changes towards positive impact vs. no – or negative impact (p < 0.05). All the “patient satisfaction” variables improved significantly from before to after therapy; Of the 8 - 18% that responded that these factors had affected their well-being negatively before treatment, 80 – 100% responded that the treatment had a positive impact (Table 4).

**Table 4:** **Impact (positive, no, or negative) of how the dental/oral health had affected the patient’s daily lives before and after treatment**

	**%Positive impact**		**% No impact**		**%Negative impact**		**% No response**	
**Patients satisfaction**	**Before after**		**Before after**		**Before after**		**Before after**	
Well being	53	80	18	11	16	3	13	7
Appearance*	43	61	35	31	11	1	11	7
Ability to chew	27	57	45	36	16	0	13	7
Personal, general mood	32	59	44	31	11	1	13	9
Social life	28	47	52	43	10	1	13	11
General health	32	53	48	37	8	0	13	11

*Appearance = Comfortability upon smiling/showing teeth.

Significant changes towards positive impact vs. no – or negative impact (p < 0.05).

All patient satisfaction variables improved significantly from before to after therapy.

## Discussion

The main finding of this study was that the patients reported improved well-being, comfortability upon smiling, ability to chew, mood, social life and general health regardless of treatment modality, which corroborates studies by Ng and Leung [10] and Wong *et al*. [20]. Thus there was no difference in reaction towards the fact that half of the participants were treated on the same day, whereas the reminder was treated over 3 weeks, indicating that the time factor was not important to them. Patient centered outcomes were improved equally in all groups [14]. It is also noteworthy that 66% of the patients in retrospect have elected to visit their dentists for maintenance more frequently than suggested by the specialist in the personally designed maintenance program. This suggests a higher dental awareness in the patients following the information and periodontal treatment, and may also explain why the oral hygiene was kept at excellent levels for the year following therapy. This very high standard of oral hygiene has been kept as the intervention study draws to a close, 5 years post-therapy (unpublished results).

The high response rate may be subscribed to these patients knowing that they participated in a scientific study, which in itself may increase the patient’s compliance and positivity to the therapy and treatment result per se [21]. Therefore, in order to reduce this impact it was important to mimic the regular clinical situation in a periodontal practice as much as possible, and not to make this a special event to the patients. The communication and clinical procedures described in this article are therefore exactly the same as the clinic and specialist provides to all periodontal patients at any time, study setting or not, and has been so since the year 2000. The total time consumption on the patients’ part was 5 – 6 hours of chair-time from first visit to the 3 months finishing consultation. At 6 and 12 months maintenance visits, with respectively the local dentist and the specialist, the chair-time would be 30 and 45 minutes, in that order.

The questionnaire was sent by surface mail to all participants to avoid delay in obtaining the answers. More importantly, a personal administered questionnaire might have caused difficulty in maintaining anonymity and it might have influenced the patients to give more positive answers than they really meant just by been handed the questionnaire by the specialist and care-provider. A surface mail delivered questionnaire was expected to reduce such bias, provide anonymity, and not be tainted with direct connection to the care provider.

Preus et al. [14] reported that most (95%) of the patients included were classified with *Severe –* [22] and 72% with *Extensive Periodontitis* [23]. However, the treatment strategies did not include surgical intervention, which by itself may have reduced anxiety and fear. It has been shown that unsubstantiated fear and anxiety towards an upcoming treatment may enhance the experience of pain and discomfort due to anxiety alone [24,25].

The strict ultimatum that the patients would not receive treatment unless their hygiene was sufficiently good may be criticized as unethical. However, the ultimatum was given together with an explanation for why this was required, and was applauded by the patients themselves. This stringency and understanding may have increased their motivation as the mean clinically obtained plaque scores were reduced substantially from screening to the 12-month visit [14]. More men (93%) than women (74%) claimed that they brushed more efficiently after treatment, contradicting other studies that find women to be more likely to accept treatment [9], and show better improvement following treatment [26]. However, the biomedical results showed that both groups improved their oral hygiene significantly, and women significantly more than men [14], indicating that men and women perceive and voice their own efforts and achievements differently.

Smoking cessation was reported differently on paper than “face – to – face” as 28 responders claimed to have quit smoking in the questionnaire. The same question was asked every patient, face to face, during the 12 months maintenance visit. However, when being faced with the specialist only 10 reported to have quit smoking. This discrepancy may reflect the difficulty to obtain true answers in assisted vs. non-assisted questionnaires, related to an often criticized and maybe personally unwanted negative and health-hazardous habit, and may indicate that such issues should be presented by assisted questionnaires and not solely on paper.

Compliance is the key factor for successful long-lasting outcome of periodontal treatment [5], but still the needed compliance is often not achieved [27,28]. Renz and Newton [28] concluded that no single behavioral model has been constructed to explain non-compliance, but suggested the need for an emotional and cognitive process to achieve a necessary level of acquiescence. Leventhal *et al*. [29] found that people will only submit to treatment if they believe that the treatment will have a positive effect on their health, and that they have the capacity to act as required. The clinical information and care package provided in this study was designed specifically based on these, and other reported concepts [2–11; 12,16], and was based on logical and comprehensive explanations and the patient’s ability to understand and succeed. The information on the etiology and pathogenesis of periodontal diseases was comprehensive and adapted to each patient’s ability to understand. The oral hygiene requirements were voiced as an ultimatum whereas the smoking information was without demands, giving the patients an opposite experience than those they were accustomed to. The patients’ worries and general dental needs were then addressed and treated, building trust ultimately followed by the periodontal treatment. The study population showed high levels of compliance and behavior change.

Perception of pain was reported low in this study, as more than 90% were satisfied with the pain reduction following therapy. A patient’s confidence with a sympathetic and understanding operator can reduce anxiety and expectancy of pain [30,31]. Moreover, Fardal and McCulloch [32] stated that for periodontal surgery and implant treatment, pain perception is affected by the level of pre-surgical anxiety, and may thus increase the perceived levels of pain experienced by the patient during treatment [24,25]. In the present study considerable efforts were made to inform and educate the patients in an empathic, albeit stringent way before treatment, in order to give true expectations to pain, treatment and results, resounding the 55% and 38% who experienced pain as - or less than expected, respectively and more than 95% being satisfied with the treatment results.

This study reported significant, positive changes after treatment in all questions regarding patient satisfaction. In this questionnaire we asked “Does teeth/dental health have an impact on your well-being, feeling comfortable upon smiling, ability to chew, mood, social life and general health before and after treatment” (positive, no impact, negative), whereas in the original instrument each item asks about the presence of a functional or psychosocial impact associated with problems involving the teeth, mouth and dentures. Our intent was to explore how much each item of dental health/disease meant to the patients, and not only the observation of function like the OHIP does. Interestingly, from one third to more than 50% of the patients already judged a positive impact from their dental health before treatment (Table 4). Many patients probably did not mind that their ability to chew was reduced or that their social life suffered from the visible or functional results of an untreated periodontal disease. They may not have recognized a change in their well-being, comfortability upon smiling, mood or general health due to periodontal disease, because it has been a slow process that have been going on for years, or they simply did not care. The suggestion to go through any treatment is motivated by the observation by a dentist or physician that a patient has diagnosed disease or condition. However, the patient’s willingness to seek treatment is also motivated by the observation that something feels wrong in his/her body, or that something is reducing his/her well-being, psychosocial aspects or function [29]. So, if such factors are unimportant to a patient, he/she will probably not seek treatment. All factors, well-being, ability to chew, comfortability upon smiling, mood, social life and general health was much more regarded with positive impact on the patients daily lives following - than prior to therapy. This suggested that the patients had become more dental aware, that they had recognized the health-effect of the treatment and that – despite recession of gums and larger interdental spaces, which in most cases was negative to their appearance - the fact that they had been treated and had achieved a state of oral health in itself seemed important to their daily lives.

In the clinically patient centered end-points, as bleeding gums and pain, there was a clear tendency towards no bleeding or pain after treatment. This was in accordance with the clinical data [14] although further comparison cannot be drawn since BOP is different from the experience of bleeding from gums provoked by hygiene performances or other.

One of the more important conclusions that may be drawn from this study was that the education the patients received regarding their own periodontal disease, treatment menu and – plan, maintenance design, and strategy to prevent recurrence, was the most important element in the information package. This was the basis for understanding why their individual hygiene -, therapy - and maintenance strategies were selected, for compliance and for motivation and skills developed. Several patients elected to respond to the questionnaires “free word comments” with statements like;” it was a relief to learn about the disease, and be told that I could take control over my own situation”.

In this respect it is disturbing to register that 72 (52%) responded to the questionnaire that they did not know that they had periodontal disease at the moment of referral. This does not mean that the dentists had not informed the patients on their situation, but rather that the patients did not understand the information properly. However, it is bestowed on the therapist to ensure that the information given to the patient is understood correctly, and therefore communication skill is of the essence.

## Conclusions

Within the limits of the present study, results indicated that patient reaction to the specific informational package and treatment resulted in a high degree of compliance; good patient centered outcomes and had a positive impact on their quality of life. The patient centered outcomes correlated in general with those clinically observed outcomes as far as a comparison could be made.

## Abbreviations

BOP: Bleeding On Probing
PD: Pocket Depth
CAL: Clinical Attachment Level
FDIS: Full Mouth Disinfection
SRP: Scaling and Root Planing
MET: Metronidazole
MI: Motivational Interviewing technique
OHIP: Oral Health Impact Profile
HELFO: The Norwegian Health Economics Administration
